# A novel and recurrent *KLHL40* pathogenic variants in a Chinese family of multiple affected neonates with nemaline myopathy 8

**DOI:** 10.1002/mgg3.1683

**Published:** 2021-05-12

**Authors:** Sheng Yi, Yue Zhang, Zailong Qin, Shang Yi, Haiyang Zheng, Jingsi Luo, Qifei Li, Jin Wang, Qi Yang, Mengting Li, Fei Chen, Qiang Zhang, Qinle Zhang, Yiping Shen

**Affiliations:** ^1^ Genetic and Metabolic Central Laboratory Birth Defect Prevention Research Institute, Maternal and Child Health Hospital, Children’s Hospital of Guangxi Zhuang Autonomous Region Nanning China; ^2^ Department of Medical Genetics and Molecular Diagnostic Laboratory Shanghai Children’s Medical Center, Shanghai Jiao Tong University School of Medicine Shanghai China; ^3^ Division of Genetics and Genomics Boston Children’s Hospital Boston MA USA; ^4^ Department of Neurology Harvard Medical School Boston MA USA

**Keywords:** appendicular hypertonia, *KLHL40*, nemaline myopathy 8, pathogenic variants, Southern Chinese

## Abstract

**Background:**

Nemaline myopathy 8 is a severe autosomal recessive muscle disorder characterized by fetal akinesia or hypokinesia, contractures, fractures, respiratory failure and swallowing difficulties apparent at birth.

**Methods:**

An affected dizygotic twin pair from a non‐consanguineous Chinese family presented with severe asphyxia, lethargy and no response to stimuli. The dysmorphic features included prominent nasal bridge, telecanthus, excessive hip abduction, limb edema, absent palmar and sole creases, acromelia, bilateral clubfoot, appendicular hypertonia and cryptorchidism. Both infants died in the first week of life. Whole‐exome sequencing was used to identify the causative gene.

**Results:**

Whole‐exome sequencing identified a recurrent missense variant c.1516A>C and a novel splice‐acceptor variant c.1153‐1G>C in *KLHL40* gene in both siblings. We estimated the disease incidence in Southern Chinese population to be 2.47/100,000 based on the cumulative allele frequency of pathogenic and likely pathogenic variants in our internal database.

**Conclusion:**

Our study expanded the mutation spectrum of *KLHL40* and the condition could have been underdiagnosed before. We identified a recurrent missense variant c.1516A>C and provided evidence further supporting the founder effect of this variant in Southern Chinese population. Given the severity of the condition and the relative high incidence, this not‐so‐rare disorder should be included in expanded carrier screening panel for Chinese population.

## Graphical abstract

Two male babies with nemaline myopathy‐8 also showed appendicular hypertonia exome sequencing identified a recurrent missense variant and a novel splice‐acceptor variant in KLHL40 Four additional novel pathogenic variants of KLHL40 were identified and it is speculated that nemaline myopathy‐8 is not‐so‐rare in Chinese population.

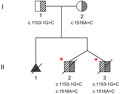

## 1 INTRODUCTION

Nemaline myopathy (NEM) is one of the most common nondystrophic congenital muscular disorders, with phenotypic variability that ranges from mild muscle dysfunction to complete akinesia and the potential for fetal death (Colombo et al., 2015). NEM is considered as a thin filament myopathy since the majority of its causative genes encode sarcomere thin filament proteins or regulators of their assembly (Garg et al., 2014). At least 12 causative genes have been recognized to date, including *ACTA1*, *CFL2*, *KBTBD13*, *KLHL40*, *KLHL41*, *LMOD3*, *MYPN*, *NEB*, *TNNT1*, *TNNT3*, *TPM2*, and *TPM3* (Sewry et al., 2019).

NEM8 (MIM #615348), caused by homozygous or compound heterozygous pathogenic variants in the *KLHL40* gene (kelch like family member 40, MIM#615340), is a severe NEM characterized by early‐onset severe generalized muscle weakness or hypokinesia, with contractures, fractures, respiratory failure, and swallowing difficulties apparent at birth. The average age of death was 5 months (Ravenscroft et al., 2013). No effective treatment is currently available for NEM patients, though it is reported that a female patient with *KLHL40*‐related NEM had a dramatic and prolonged improvement after treatment with acetylcholinesterase inhibitors (Natera‐de Benito et al., 2016).

To date, more than 40 patients with NEM8 have been reported in different regions of the world. And 33 mutations in *KLHL40* gene have been identified so far (Table S1). Here, we reported two neonates with NEM caused by compound heterozygous variants in the *KLHL40* gene from a non‐consanguineous Chinese family. The patients presented common features of *KLHL40*‐related NEM and also showed appendicular hypertonia. Our report expanded the clinical phenotype and genetic variation spectrum of *KLHL40*.

Besides a novel splicing variant in the patients, we identified four additional novel pathogenic variants of *KLHL40* by screening all variants in our in‐house database. The expanded mutation spectrum of the *KLHL40* gene would improve the diagnosis of NEM8. Although only one such family was observed so far, by estimating the total allele frequency (AF) of pathogenic and likely pathogenic variants of *KLHL40*, we speculated that the condition is not‐so‐rare in the local population and the gene/variants should be included in expanded carrier screening panel.

## 2 MATERIALS AND METHODS

### 2.1 Patients

A total of 2866 participants (1104 patients and 1762 controls) were enrolled from January, 2016 to December, 2019 in the Maternal and Child Health Hospital of Guangxi Zhuang Autonomous Region.

### 2.2 Molecular analysis

Genomic DNA was isolated from peripheral blood lymphocytes using Lab‐Aid DNA kit (Zeesan Biotech Co., Ltd, Xiamen, China). For whole‐exome sequencing, genomic DNA samples were captured to create a sequencing library by Agilent SureSelect Human All Exon V5 Kit (Agilent Technologies, Santa Clara, CA), and the prepared libraries were sequenced on a HiSeq2500 (Illumina, San Diego, CA). Sequence alignment and variant calling against the reference human genome (GRCh37) were performed using BWA and the Genome Analysis Toolkit (GATK HaplotypeCaller; McKenna et al., 2010). Copy number variants (CNV) analysis based on read depth method was performed using an in‐house pipeline, and CNVs of significant interest were further visually inspected with the Integrative Genomics Viewer. Single nucleotide variants and indels were annotated and prioritized by the TGex software (LifeMap Sciences, Alameda, CA). The variant pathogenicity was assessed according to American College of Medical Genetics and Genomics/Association for Molecular Pathology (ACMG/AMP) guidelines (Richards et al., 2015). All the potentially causative variants were verified by polymerase chain reaction and Sanger sequencing. All operations were carried out according to the instructions of the manufacturers. The variants were described by the accession number NM_152393.3 for *KLHL40*. The bioinformatic splicing tool Human Splicing Finder (HSF) version 3.0 (http://www.umd.be/HSF3/) was applied to predict the possible influence of variation near intron/coding exon boundary on pre‐mRNA splicing.

## 3 RESULTS

### 3.1 Clinical description

**FIGURE 1:**
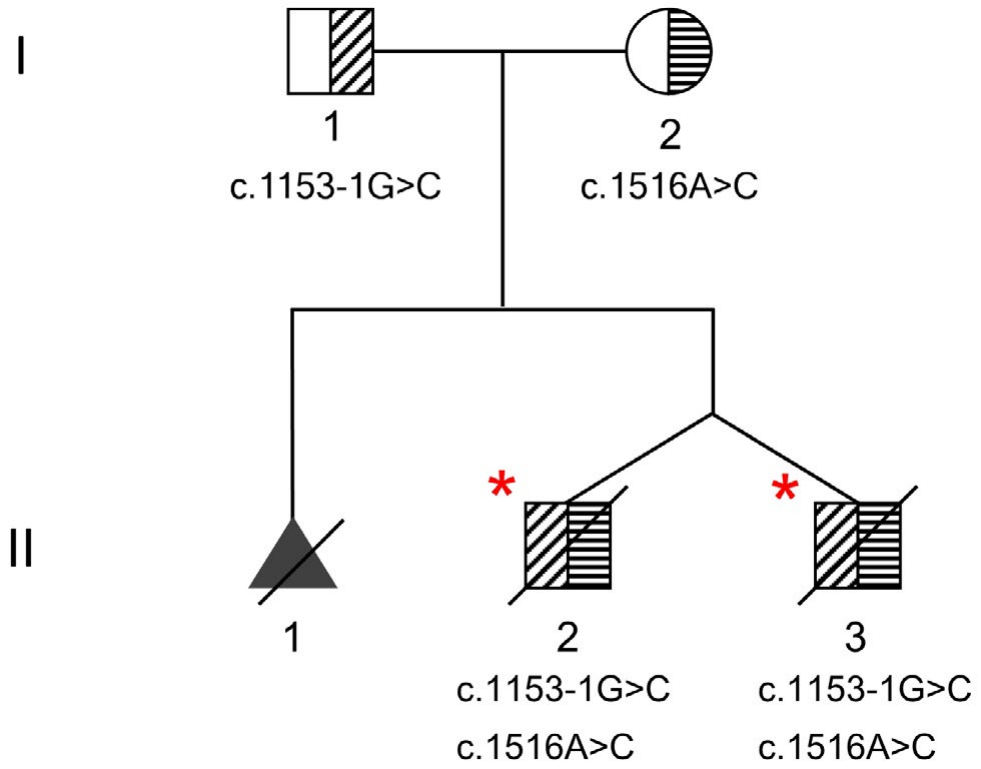
The family pedigree (upper part) and DNA sequencing of the variants (lower part). Asterisks indicate the probands. Unaffected parents are both carriers. The symptomatic fetus was not genetically verified

The two male siblings (dizygotic twin) were from a healthy, non‐consanguineous, Chinese couple (Figure 1). The patients’ mother (gravida 2, para 1) had previously terminated a pregnancy due to fetal akinesia. Ultrasound studies of the dichorionic twins during pregnancy revealed increased nuchal translucency thickness and fetal tachycardia. Single nucleotide polymorphism (SNP). array results of chorionic villus sampling were both normal. They were born at 37 weeks of gestation. The elder brother had a weight of 2540 g, head circumference of 34 cm, Apgar scores at 1 and 5 min were 3 and 5, respectively. The younger brother had a weight of 2160 g, head circumference of 36 cm, Apgar scores at 1 and 5 min were 3 and 4, respectively. They did not cry at birth and both had the clinical features as follows: severe asphyxia, appendicular hypertonia, lethargy, no stimulus response, hypertelorism, telecanthus, prominent nasal bridge, excessive hip abduction, tetraphocomelia, limbs edema, bilateral talipes equinovarus, absent palmar crease, undescended testes. In addition, the younger brother had skin ecchymosis which was absent from the elder brother. Both infants died within a week after birth.

### 3.2 Genetic analysis

**FIGURE 2:**
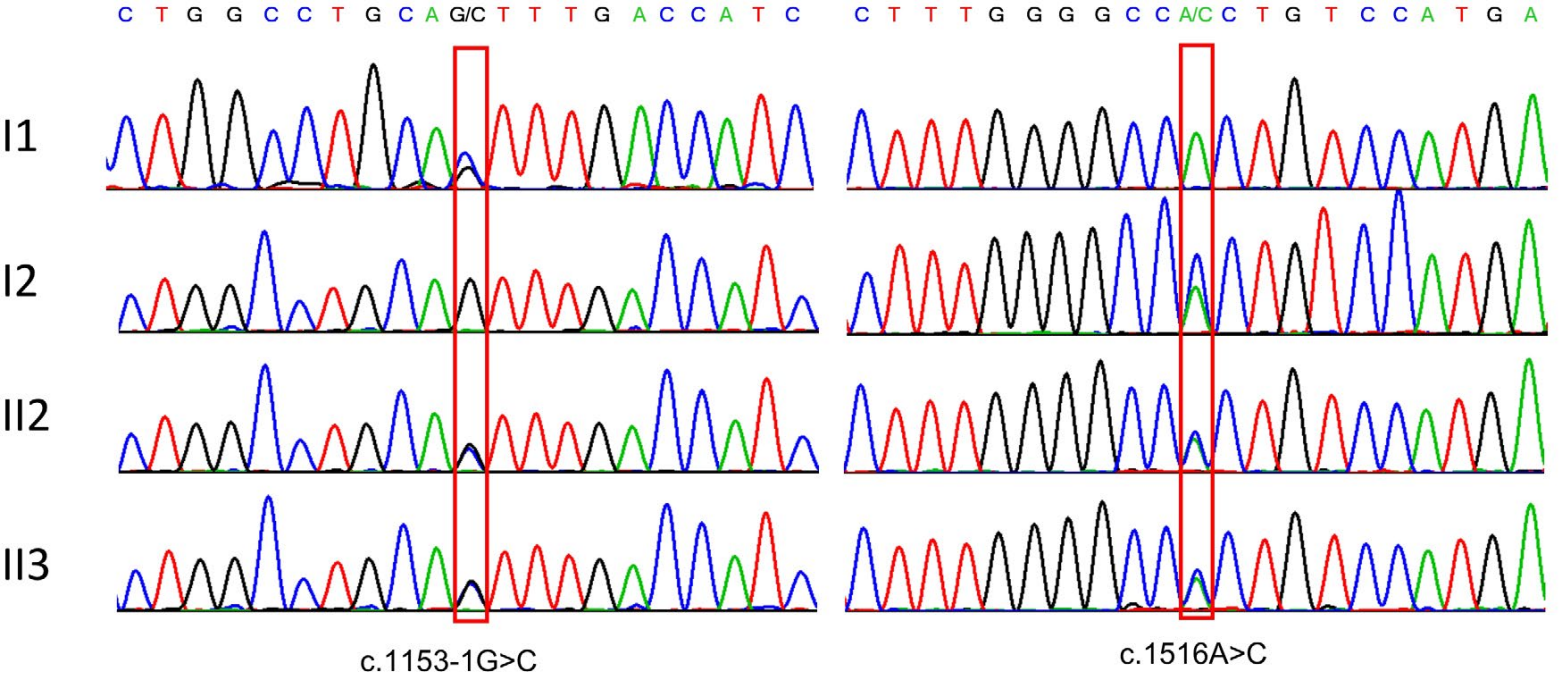
Variants identification by Sanger sequencing. The red frame represents the variant sites (c.1153‐1G and c.1516A, respectively)

Trio‐based whole‐exome sequencing revealed compound heterozygous variants, c.1153‐1G>C and c.1516A>C p.(Thr506Pro), in the *KLHL40* gene (NM_152393.3) in both siblings (Figure 2). Both asymptomatic parents were heterozygous carriers. The paternally inherited splice‐site variant is novel, and it is predicted to break the original splicing site by the bioinformatic splicing tool Human Splicing Finder (http://www.umd.be/HSF3/) (Table S2). The maternally inherited missense variant was previously reported in affected individuals, a known founder mutation in ethnic Chinese (Lee et al., 2019). Both variants can be classified as pathogenic following the ACMG/AMP guidelines (Richards et al., 2015) and the evidence were shown in Table S3.

## 4 DISCUSSION

*KLHL40* gene contains 6 coding exons, encodes a 621 amino‐acid protein, including an N‐terminal BTB domain which function as substrate‐specific adaptors for Cullin‐3 (Cul3), a BACK motif, and a kelch repeat (Blondelle et al.,^2019^; Garg et al., 2014; Gupta & Beggs, 2014). KLHL40 protein is a member of the BTB/Kelch adaptor protein family which are involved in a plethora of molecular and cellular mechanisms, such as cell migration, morphology, and protein expression (Gupta & Beggs, 2014). As a stabilizer of the thin filament proteins leiomodin‐3 and nebulin, KLHL40 is necessary for both myogenesis and skeletal‐muscle maintenance (Garg et al., 2014).

**FIGURE 3:**
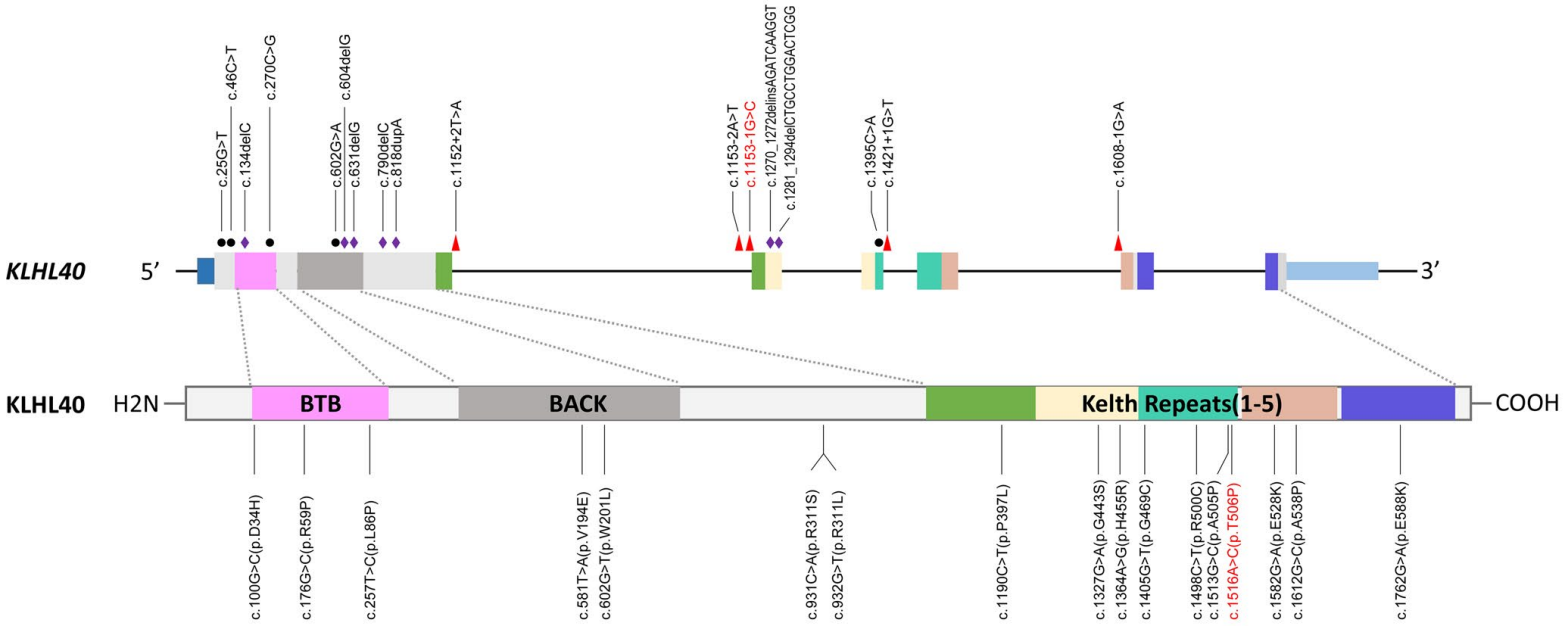
The spectrum of *KLHL40* mutations. The schematic presentation of the genomic structure of the *KLHL40* gene (upper) and the corresponding encoded protein domains (lower) are drawn in scale. Loss‐of‐function mutations are shown above the *KLHL40* gene. Nonsense mutations are indicated by black dots. Frameshift mutations are marked with purple diamonds. Splicing mutation are depicted by red triangles. Missense mutations are shown below the schematic diagram of protein domain structures. The novel variant (c.1153‐1G>C) and the recurrent variant (c.1516A>C) in our patients are highlighted in red

NEM8 is an autosomal‐recessive neuromuscular disorder caused by diverse mutations throughout the *KLHL40* gene (Figure 3). As was reported, the majority of patients with NEM8 showed prenatal symptoms (83%). Respiratory failure (96.6%), dysphagia (95.8%), and contractures (88.9%) were noted at neonatal period in most patients. In addition, almost all affected individuals had facial involvement and muscle weakness (Ravenscroft et al., 2013). In the present report, the clinical phenotypes of the affected twins were consistent with previous reports, including fetal edema, contractures, respiratory failure and mild dysmorphology. However, the twins also showed appendicular hypertonia, which is in contrast to what was reported previously. It is regrettable that the twins both died in the first week of life. We were not able to acquire more detailed clinical information, and the muscle pathology was not assessed.

The splice‐acceptor variation, of *KLHL40* gene, c.1153‐1G>C, is not listed in the SNP database, ClinVar or the human gene mutation database. And it was absent from general population. It is a canonical splicing variant. The HSF tool showed that the acceptor site score of the variant decreased significantly (−33.53%) and it is suspected that the mutant sequence may have broken the highly conserved AG dinucleotides of the splice junction (Table S2). Meanwhile, analysis of exonic splicing enhancer and exonic splicing silencer showed the transversion would break the reference motif, respectively (Table S2). However, its pathological effect was not further confirmed by functional analysis.

The missense variant, c.1516A>C p.(Thr506Pro), was listed in the dbSNP database (rs778022582). The Thr506 residue is located in the kelch‐repeat domain of KLHL40, and the substitution would destabilize the intramolecular interactions of the β sheet and thus impair protein stability (Ravenscroft et al., 2013). According to gnomAD, it is only distributed in East Asian with a relatively high AF of 0.0013. Five patients with the missense variant had been previously reported, three patients with homozygous c.1516A>C and the other two with compound heterozygous variants (Lee et al., 2019; Ravenscroft et al., 2013). Besides these patients, c.1516A>C was identified in two affected fetuses from one family with multiple joint contractures aborted electively at 21 and 28 weeks, respectively (Chen et al., 2016). These patients are all from unrelated Chinese families. Lee et al. had demonstrated the founder effect of c.1516A>C p.(Thr506Pro) by polymorphic marker analysis and the variant might have occurred around 412 generations ago (Lee et al., 2019).

In order to assess the AF of pathogenic variants, we reviewed all the detected variants in *KLHL40* gene in our in‐house database (*n* = 2866). After filtering out the variants with AF >1% or located in the non‐coding regions, 54 variations remained (Table S4). According to the ACMG/AMP guidelines, three variants are pathogenic (one novel) and three are likely pathogenic (all novel). The overall AF in our cohort was estimated to be nearly 0.0031, thus the predicted incidence of NEM caused by *KLHL40* variants would be 2.47/10^6^ in Southern China. This could be an underestimate since some missense variants of uncertain significance could eventually be classified as pathogenic. The recurrent pathogenic variant (c.1516A>C) has a local AF of 0.14%, which is very close to the AF reported for East Asian (0.13%), indicating this condition may have been under detected across the country, due to a lack of clinical and molecular diagnosis in most cases.

In conclusion, we reported two male babies with NEM8 from a non‐consanguineous Chinese family. Besides the common features of *KLHL40*‐associated NEM, the patients also presented a distinct phenotype. Our study expanded the mutation spectrum of *KLHL40* and the condition could have been underdiagnosed before. Given the severity of the condition and the relative high incidence, this not‐so‐rare disorder should be included in expanded carrier screening panel for Chinese population.

## ETHICS APPROVAL

This work was approved by the Ethics Committee of Maternal and Child Health Hospital of Guangxi Zhuang Autonomous Region.

## INFORMED CONSENT

Informed consent was collected from the family.

## CONFLICT OF INTEREST

The authors declare no conflict of interest.

## AUTHOR CONTRIBUTIONS

Collection of clinical data: Yue Zhang and Jingsi Luo. Data analyzed and interpreted: Sheng Yi, Zailong Qin, Shang Yi, Haiyang Zheng, Qifei Li, Jin Wang, Qi Yang, Mengting Li, Fei Chen, Qiang Zhang, Qinle Zhang. Writing and review of original draft of the manuscript: Sheng Yi and Yiping Shen.

## Supporting information

Table S1

Table S2

Table S3

Table S4

## Data Availability

All data generated or analyzed during this study are included in this published article and its supporting information files.
