# Supplementary material for: A novel and recurrent KLHL40 pathogenic variants in a Chinese family of multiple affected neonates with nemaline myopathy 8
Source: Mol Genet Genomic Med. 2021 May 12;9(6):e1683. doi: 10.1002/mgg3.1683 (PMC8222828; doi:10.1002/mgg3.1683)
Supplement: Supplementary file 1 — Table S1 [file MGG3-9-e1683-s002.docx]

Table S1. Information of in *KLHL40* mutations have been identified.

| **Location** | **Nucleotide change** † | **Amino-acid change** | **Mutation Type** | **Reference** |
| --- | --- | --- | --- | --- |
| Exon 1 | c.25G>T | p.(Glu9*) | Nonsense | ClinVar: VCV000804436 |
| Exon 1 | c.46C>T | p.(Gln16*) | Nonsense | PubMed: Todd 2015 |
| Exon 1 | [c.100G>C](https://databases.lovd.nl/shared/variants/0000128529#00023995) | p.(Asp34His) | Missense | PubMed: Ravenscroft 2013 |
| Exon 1 | [c.134del](https://databases.lovd.nl/shared/variants/0000128530#00023995) | p.(Pro45Argfs*19) | Frameshift | PubMed: Ravenscroft 2013 |
| Exon 1 | c.176G>C | p.(Arg59Pro) | Missense | ClinVar: VCV000807621 |
| Exon 1 | [c.257T>C](https://databases.lovd.nl/shared/variants/0000128561#00023995) | p.(Leu86Pro) | Missense | PubMed: Ravenscroft 2013 |
| Exon 1 | [c.270C>G](https://databases.lovd.nl/shared/variants/0000128531#00023995) | p.(Tyr90*) | Nonsense | PubMed: Ravenscroft 2013 |
| Exon 1 | [c.581T>A](https://databases.lovd.nl/shared/variants/0000128532#00023995) | p.(Val194Glu) | Missense | PubMed: Ravenscroft 2013 |
| Exon 1 | [c.602G>A](https://databases.lovd.nl/shared/variants/0000128534#00023995) | p.(Trp201*) | Nonsense | PubMed: Ravenscroft 2013 |
| Exon 1 | [c.602G>T](https://databases.lovd.nl/shared/variants/0000128533#00023995) | p.(Trp201Leu) | Missense | PubMed: Ravenscroft 2013 |
| Exon 1 | c.604delG | p.(Ala202Argfs*56) | Frameshift | PubMed: Natera-de Benito 2016 |
| Exon 1 | c.631delG | p.(Ala211Leufs*47) | Frameshift | ClinVar: VCV000658674 |
| Exon 1 | [c.790del](https://databases.lovd.nl/shared/variants/0000128535#00023995)C | p.(Arg264Alafs*59) | Frameshift | PubMed: Ravenscroft 2013 |
| Exon 1 | c.818dupA | p.( Lys275Glufs*10) | Frameshift | ClinVar: VCV000650910 |
| Exon 1 | [c.931C>A](https://databases.lovd.nl/shared/variants/0000128559#00023995) | p.(Arg311Ser) | Missense | PubMed: Todd 2015 |
| Exon 1 | [c.932G>T](https://databases.lovd.nl/shared/variants/0000128536#00023995) | p.(Arg311Leu) | Missense | PubMed: Ravenscroft 2013 |
| Intron 1 | c.1152+2T>A | NA | Splice donor | ClinVar: VCV000653053 |
| Intron 1 | c.1153-2A>T | NA | Splice acceptor | ClinVar: VCV000541330 |
| Exon 2 | [c.1190C>T](https://databases.lovd.nl/shared/variants/0000128537#00023995) | p.(Pro397Leu) | Missense | PubMed: Ravenscroft 2013 |
| Exon 2 | [c.1270_1272delinsAGATCAAGGT](https://databases.lovd.nl/shared/variants/0000128538#00023995) | p.(Asp424Argfs*23) | Frameshift | PubMed: Ravenscroft 2013 |
| Exon 2 | [c.1281_1294del](https://databases.lovd.nl/shared/variants/0000128539#00023995) | p.(Cys428Hisfs*12) | Frameshift | PubMed: Ravenscroft 2013 |
| Exon 3 | c.1327G>A | p.(Gly443Ser) | Missense | PubMed: Lee 2019 |
| Exon 3 | [c.1364A>G](https://databases.lovd.nl/shared/variants/0000128540#00023995) | p.(His455Arg) | Missense | PubMed: Ravenscroft 2013 |
| Exon 3 | c.1395C>A | p.(Tyr465*) | Nonsense | ClinVar: VCV000541327 |
| Exon 3 | [c.1405G>T](https://databases.lovd.nl/shared/variants/0000128541#00023995) | p.(Gly469Cys) | Missense | PubMed: Ravenscroft 2013 |
| Intron 3 | [c.1421+1G>T](https://databases.lovd.nl/shared/variants/0000128567#00023995) | NA | Splice donor | ClinVar: VCV000571143 |
| Exon 4 | c.1498C>T | p.(Arg500Cys) | Missense | PubMed: Seferian 2016 |
| Exon 4 | c.1513G>C | p.(Ala505Pro) | Missense | PubMed: Natera-de Benito 2016 |
| Exon 4 | [c.1516A>C](https://databases.lovd.nl/shared/variants/0000128562#00023995) | p.(Thr506Pro) | Missense | PubMed: Ravenscroft 2013 |
| Exon 4 | [c.1582G>A](https://databases.lovd.nl/shared/variants/0000128543#00023995) | p.(Glu528Lys) | Missense | PubMed: Ravenscroft 2013 |
| Intron 4 | [c.1608-1G>A](https://databases.lovd.nl/shared/variants/0000128555#00023995) | NA | Splice acceptor | PubMed: Ravenscroft 2013 |
| Exon 5 | [c.1612G>C](https://databases.lovd.nl/shared/variants/0000128556#00023995) | p.(Ala538Pro) | Missense | PubMed: Ravenscroft 2013 |
| Exon 6 | [c.1762G>A](https://databases.lovd.nl/shared/variants/0000128563#00023995) | p.(Glu588Lys) | Missense | PubMed: Ravenscroft 2013 |

† Numbering for DNA mutation is based on cDNA sequence (GenBank no. NM_152393.3), with nucleotide +1 corresponding to A of the ATG translation initiation codon.
