# Supplementary material for: A novel and recurrent KLHL40 pathogenic variants in a Chinese family of multiple affected neonates with nemaline myopathy 8
Source: Mol Genet Genomic Med. 2021 May 12;9(6):e1683. doi: 10.1002/mgg3.1683 (PMC8222828; doi:10.1002/mgg3.1683)
Supplement: Supplementary file 2 — Table S2 [file MGG3-9-e1683-s003.docx]

**Table S2. In-silico predictions for 3’ acceptor splice sites using HSF, exonic splicing enhancer, and exonic splicing silencer**

| **cDNA position** | **Type** | **Reference motif** | **Mutant motif** | **Score (0–100) WT/MU** | **Variation (%)** | **Interpretation** |
| --- | --- | --- | --- | --- | --- | --- |
| -12 | Acceptor | cctggcctgcagTT | cctggcctgcacTT | 86.34/57.39 | -33.53 | WT SS broken |
| -4 | Donor | gcagTTTGA | GCActttga | 66.75/39.91 | -40.21 | WT SS broken |
| -6 | SF2/ASF | ctgcagT | - | 71.92/- | -100 | Breaks the ESE |
| -5 | - | tgcagT | - | - | - | Breaks the ESS |
| -3 | - | cagTTT | - | - | - | Breaks the ESS |
| -2 | - | agTTTG | - | - | - | Breaks the ESS |

Consequences were predicted using the HSF prediction algorithm. ESE, exonic splicing enhancer; ESS, exonic splicing silencer; HSF, Human Splicing Finder; SF2/ASF, a splicing factor; SS, splice site; WT/MU, wild-type and mutated sequences.
