# Supplementary material for: A novel and recurrent KLHL40 pathogenic variants in a Chinese family of multiple affected neonates with nemaline myopathy 8
Source: Mol Genet Genomic Med. 2021 May 12;9(6):e1683. doi: 10.1002/mgg3.1683 (PMC8222828; doi:10.1002/mgg3.1683)
Supplement: Supplementary file 3 — Table S3 [file MGG3-9-e1683-s004.docx]

**Table S3. Details of pathogenic identiﬁed variants of*KLHL40***

| **Variants** | **Variants Type** | **Exon/Intron** | **In silico prediction** | | | **ACMG/AMP classification** |
| --- | --- | --- | --- | --- | --- | --- |
|  |  |  | SIFT | PolyPhen-2 | Mutation Taster |  |
| c. 1153-1G>C | splice-acceptor | Intron_1 | NA | NA | disease causing | PVS1+PM2+PM3 (P) |
| c.1516A>C (p.Thr506Pro) | missense | Exon_4 | damaging | possibly damaging | disease causing | PM1+  PM3_strong+  PS3 (P) |

Nucleotide numbering is based on NM_152393.3. Classification of in silico prediction: SIFT, damaging or tolerated; PolyPhen-2, probably damaging, possibly damaging, or benign; MutationTaster, polymorphism or disease causing; NA, not available. ACMG/AMP classification: PVS1: null variant; PM2: Absence of the variant from controls (or at extremely low frequency if recessive) in publicly available databases; PM3: For recessive disorders, detected in trans with a pathogenic variant; PM1: Located in a mutational hot spot and/or critical and well-established functional domain; PS3: Well-established in vitro or in vivo functional studies supportive of a damaging effect; P: pathogenic; LP: likely pathogenic.
