# Supplementary material for: A novel and recurrent KLHL40 pathogenic variants in a Chinese family of multiple affected neonates with nemaline myopathy 8
Source: Mol Genet Genomic Med. 2021 May 12;9(6):e1683. doi: 10.1002/mgg3.1683 (PMC8222828; doi:10.1002/mgg3.1683)
Supplement: Supplementary file 4 — Table S4 [file MGG3-9-e1683-s001.docx]

**Table S4 Estimated Pathogenicity of the detected *KLHL40* variants in our local database** **on ACMG guidelines**

| **Position** | **Nucleotide**  **change** | **Amino acid**  **change** | **No. of Het** | | **Clinical manifestations of variant carriers** | **AF local** | **Pathogenicity** |
| --- | --- | --- | --- | --- | --- | --- | --- |
|  |  |  | **Controls** | **Patients** |  |  |  |
| chr3:42727208 | c.98T>C | p.(Leu33Pro) | 1 | 1 | AM | 0.00034892 | VUS |
| chr3:42727234 | c.124C>A | p.(Arg42Ser) | 1 | 1 | SS | 0.00034892 | VUS |
| chr3:42727319 | c.209G>C | p.(Gly70Ala) | 1 | 0 |  | 0.00017446 | VUS |
| chr3:42727352 | c.242T>C | p.(Val81Ala) | 1 | 0 |  | 0.00017446 | VUS |
| chr3:42727362 | c.252G>A | p.(Gln84Gln) | 1 | 1 |  | 0.00034892 | B |
| chr3:42727387 | c.277G>C | p.(Glu93Gln) | 13 | 17 |  | 0.00523378 | B |
| chr3:42727402 | c.292G>A | p.(Glu98Lys) | 1 | 1 | SS | 0.00034892 | VUS |
| chr3:42727471 | c.361G>C | p.(Val121Leu) | 0 | 1 | SS | 0.00017446 | VUS |
| chr3:42727515 | c.405C>T | p.(Ala135Ala) | 0 | 1 |  | 0.00017446 | B |
| chr3:42727516 | c.406G>A | p.(Val136Ile) | 1 | 0 |  | 0.00017446 | VUS |
| chr3:42727539 | c.429C>A | p.(Leu143Leu) | 0 | 4 |  | 0.00069784 | B |
| chr3:42727540 | c.430G>T | p.(Asp144Tyr) | 0 | 1 | SS | 0.00017446 | VUS |
| chr3:42727547 | c.437C>T | p.(Ala146Val) | 0 | 1 | SS | 0.00017446 | VUS |
| chr3:42727582 | c.472G>T | p.(Ala158Ser) | 0 | 1 | SS | 0.00017446 | VUS |
| **chr3:42727712** | **c.602G>A** | **p.(Trp201*)** | **1** | **2** | **MW / GDD** | **0.00052338** | **P(PVS1+PM2+PM3_supporting)** |
| chr3:42727763 | c.653C>T | p.(Pro218Leu) | 1 | 0 |  | 0.00017446 | VUS |
| chr3:42727829 | c.719G>A | p.(Arg240His) | 1 | 1 | SS | 0.00034892 | VUS |
| chr3:42727883 | c.773A>C | p.(Lys258Thr) | 1 | 2 | SS / SS | 0.00052338 | VUS |
| chr3:42727974 | c.864C>T | p.(Ser288Ser) | 3 | 5 |  | 0.00139567 | B |
| chr3:42728022 | c.912G>A | p.(Gly304Gly) | 2 | 2 |  | 0.00069784 | B |
| chr3:42728056 | c.946C>T | p.(Leu316Leu) | 1 | 0 |  | 0.00017446 | B |
| chr3:42728101 | c.991T>C | p.(Tyr331His) | 1 | 2 | GDD / GDD | 0.00052338 | VUS(PP3) |
| chr3:42728164 | c.1054G>C | p.(Val352Leu) | 0 | 1 | SS | 0.00017446 | VUS |
| chr3:42728193 | c.1083C>T | p.(Val361Val) | 1 | 0 |  | 0.00017446 | B |
| chr3:42728197 | c.1087G>C | p.(Val363Leu) | 0 | 1 | SS | 0.00017446 | VUS |
| chr3:42728214 | c.1104C>T | p.(Phe368Phe) | 1 | 1 |  | 0.00034892 | B |
| chr3:42728240 | c.1130C>T | p.(Pro377Leu) | 1 | 0 |  | 0.00017446 | VUS |
| chr3:42729631 | c.1153-3C>T | NA | 1 | 1 | GDD | 0.00034892 | VUS |
| **chr3:42729633** | **c.1153-1G>C** | **NA** | **1** | **2** | **NM / NM** | **0.00052338** | **P(PVS1+PM2+PM3)** |
| **chr3:42729652** | **c.1171G>T** | **p.(Glu391*)** | **0** | **1** | **SS** | **0.00017446** | **LP(PVS1+PM2)** |
| chr3:42729670 | c.1189C>A | p.(Pro397Thr) | 0 | 1 | SS | 0.00017446 | VUS |
| chr3:42729733 | c.1252G>A | p.(Gly418Ser) | 1 | 1 | SS | 0.00034892 | VUS(PP3) |
| chr3:42729760 | c.1279C>T | p.(Arg427Cys) | 1 | 0 |  | 0.00017446 | VUS |
| chr3:42729773 | c.1292C>T | p.(Ser431Leu) | 0 | 3 | GDD / GH / SS | 0.00052338 | VUS |
| chr3:42730123 | c.1335G>A | p.(Ser445Ser) | 1 | 2 |  | 0.00052338 | B |
| chr3:42730129 | c.1341G>A | p.(Pro447Pro) | 0 | 1 |  | 0.00017446 | B |
| chr3:42730175 | c.1387C>A | p.(Leu463Ile) | 0 | 1 | GDD | 0.00017446 | VUS |
| chr3:42730366 | c.1427G>A | p.(Cys476Tyr) | 0 | 1 | AM | 0.00017446 | VUS |
| chr3:42730416 | c.1477C>G | p.(Leu493Val) | 2 | 3 | SE / GH / SS | 0.0008723 | VUS |
| **chr3:42730438** | **c.1499G>A** | **p.(Arg500His)** | **0** | **1** | **MW** | **0.00017446** | **LP(PM1+PM2+PM5+PP3)** |
| **chr3:42730455** | **c.1516A>C** | **p.(Thr506Pro)** | **2** | **6** | **GDD / NM / NM / SS / SS / SS** | **0.00139567** | **P(PM1_strong,** **PM3_strong, PS3)** |
| chr3:42730530 | c.1591A>G | p.(Ser531Gly) | 0 | 1 | SS | 0.00017446 | VUS |
| **chr3:42732355** | **c.1612G>A** | **p.(Ala538Thr)** | **1** | **1** | **GH** | **0.00034892** | **LP(PM1+PM2+PM5)** |
| chr3:42732363 | c.1620C>T | p.(Phe540Phe) | 1 | 1 |  | 0.00034892 | B |
| chr3:42732379 | c.1636G>C | p.(Glu546Gln) | 3 | 5 | GDD / GH / SE / SS / SS | 0.00139567 | VUS |
| chr3:42732428 | c.1685T>C | p.(Ile562Thr) | 1 | 1 | SS | 0.00034892 | VUS |
| chr3:42732476 | c.1733C>G | p.(Thr578Arg) | 0 | 1 | SS | 0.00017446 | VUS |
| chr3:42732481 | c.1738C>A | p.(Leu580Ile) | 2 | 1 |  | 0.00052338 | LB |
| chr3:42733374 | c.1755G>A | p.(Arg585Arg) | 2 | 1 |  | 0.00052338 | B |
| chr3:42733381 | c.1762G>A | p.(Glu588Lys) | 7 | 11 |  | 0.00314027 | LB |
| chr3:42733443 | c.1824C>A | p.(Phe608Leu) | 0 | 1 | AM | 0.00017446 | VUS |
| chr3:42733444 | c.1825C>G | p.(Leu609Val) | 0 | 1 | AM | 0.00017446 | VUS |
| chr3:42733451 | c.1832T>A | p.(Val611Glu) | 0 | 1 | AM | 0.00017446 | VUS |
| chr3:42733465 | c.1846C>A | p.(Leu616Met) | 3 | 8 | GDD / GH / SE / AM / SS / SS / SS | 0.00191905 | VUS |

Numbering for DNA variant is based on cDNA sequence (GenBank no. NM_152393.3). AF: allele frequency; VUS: uncertain significance; LB: likely benign; B: benign; LP: likely pathogenic; P: pathogenic; Het: heterozygous. PVS1: null variant; PS3: Well-established in vitro or in vivo functional studies supportive of a damaging effect; PM1: Located in a mutational hot spot and/or critical and well-established functional domain; PM2: Absence of the variant from controls (or at extremely low frequency if recessive) in publicly available databases; PM3: For recessive disorders, detected in trans with a pathogenic variant; PM5: Novel missense change at an amino acid residue where a different missense change determined to be pathogenic has been seen before; PP3: Multiple lines of computational evidence support a deleterious effect. The published mutations are depicted in bright yellow, and the novel pathogenic or likely pathogenic variants are highlighted in light green. The clinical manifestations of the variant carriers are appended after the numbers (Information on benign or likely benign variants are not provided). AM: Abnormality of metabolism; SS: Short stature; MW: Muscle weakness; GDD: Global developmental delay; NM: Nemaline myopathy; GH: Gonadal hypoplasia; SE: Seizures.
